# Spatial Partitioning of miRNAs Is Related to Sequence Similarity in Overall Transcriptome

**DOI:** 10.3390/ijms17060830

**Published:** 2016-06-08

**Authors:** William Seffens, Fisseha Abebe, Chad Evans, Xiao-Qian Wang

**Affiliations:** 1 Physiology Department, Morehouse School of Medicine, Atlanta, GA 30310, USA; crevans@msm.edu; 2 Mathematics Department, Clark Atlanta University, Atlanta, GA 30314, USA; fabebe2014@gmail.com; 3 Physics Department, Clark Atlanta University, Atlanta, GA 30314, USA; xwang@cau.edu

**Keywords:** miRNA, transcriptome, exosome

## Abstract

RNAs have been shown to exhibit differential enrichment between nuclear, cytoplasmic, and exosome fractions. A current fundamental question asks why non-coding RNA partition into different spatial compartments. We report on the analysis of cellular compartment models with miRNA data sources for spatial-mechanistic modeling to address the broad area of multi-scalar cellular communication by miRNAs. We show that spatial partitioning of miRNAs is related to sequence similarity to the overall transcriptome. This has broad implications in biological informatics for gene regulation and provides a deeper understanding of nucleotide sequence structure and RNA language meaning for human pathologies resulting from changes in gene expression.

## 1. Introduction

Much focus in biology is directed toward explaining the regulation of protein-coding genes, but lately, interaction networks with non-coding RNAs (ncRNAs) have been under particular scrutiny [1]. There is a suggestion that broad communication networks concerning competitive endogenous RNAs (ceRNAs) exist whereby ncRNAs could modulate regulatory RNA by binding and titrating from sites of protein-coding messenger RNAs [2]. Generally, RNA molecules and proteins undergo constrained diffusion largely limited by spatial constraints of other molecules and move by a stop-and-go mechanism where free diffusion is interrupted by random association and collision with other cellular structures [3]. Most importantly, the dynamic nature of RNAs is emerging as a means to control physiologic cellular responses and pathways [4]. Brownian motion effects are ubiquitous and play a pivotal role when one infers macroscopic functions from the mesoscopic level of description, a route commonly utilized in the study of complex systems. Dynamics at such mesoscopic level is dictated by a set of Langevin processes or equivalently by the associated N-particle Fokker–Planck equation [5]. We apply that conceptual model basis in the present work to examine miRNAs diffusing in a fluid medium that exhibits global RNA interaction resulting from nucleotide motifs or sequence words. We seek to determine if miRNAs with sequence words in common with the whole transcriptome have enhanced mobility since their transport can be facilitated by common transcript pathways, and, due to their small size of 22 nucleotides (nt), could have an influence on transport to the extracellular space, in the form of exosomes.

## 2. Results

### 2.1. Whole Transcriptome as an Information Cloud of Sequence Words

We propose that miRNA localization in cellular compartments is an emergent property from interactions of a cloud of RNA-binding proteins and RNA sequences composed of nucleotide words. Here, words are extracted from sliding windows over all transcript sequences with some functional window size. An emergent consequence of this cloud model is that anomalous diffusion can occur if random-walk target RNA transcripts interact with surrounding protein scaffold as a cloud, and if the cloud relaxation time is long [6]. This would be similar to what can be observed with falling objects clustering or trailing in a fluid [7]. We propose that RNAs with sequences similar to the whole transcriptome will exhibit enhanced transport compared to RNA sequences without similar sequences. Thus, miRNAs should partition into different cellular compartments based on word compositions from their sequence. We can determine the frequency of all words in the transcriptome as a matrix composed of RNA sequences and copy levels. For each transcript, we count the number of words in common with all others in the cloud list or dictionary as a similarity measure to the transcriptome, and we were also able to compare them to randomized sequence words.

RNA molecule diffusion initially in nuclear then cytoplasmic compartments would lead to extracellular export of RNA if the transcript half-life is greater than its transit rate. Calculations at arbitrary transit distances could be determined from a dynamic systems model with a large set of partial differential equations modeling RNA mobility as in the Fick’s equation, but this would be computationally prohibitive [8]. Instead, we pursue a thermodynamic approach based on the Fokker–Planck equation [5]. Consider that each transcript is affected by local protein scaffolds with an effective interaction window of some sequence length w. The closer the word set of the target (miRNA) to the whole transcriptome, the more canonical its diffusion. As such, the mobility displays consistent patterns with the whole transcriptome. Anomalous RNA diffusion can give rise to emergent and patterned behavior in the cell [9]. Some transcripts will have specialized transport modes, which will show up as outliers in this algorithmic treatment. The transcriptome cloud dictionary is built as a collection of transcriptome word sets along with expression levels that depend on the cell state. Model parameters like optimum word size can be estimated from RNA datasets obtained from public data sources. Assume that the smallest reasonable word in the cloud is four nt long, this corresponding to the lower limit of size for a seed sequence in miRNA [10]. In this case, there are only 4^4^ = 256 different words so that the transcriptome dictionary would have high expression values for the many duplicate words. The upper limit for word size is set at 22 nt, corresponding to the size of a typical mature miRNA. This is the same as the MRE size in the similarly related ceRNA hypothesis by [11]. We determine the frequency of all words in the transcriptome with a matrix composed of RNA sequences and copy levels (e.g., normalized reads or RPKM). For each miRNA target transcript, count the number of words in common with the cloud dictionary as a similarity measure (“tCount”) to the transcriptome, or multiply each word count (tCount) by its expression value to derive “tWord” measure.

The maximal size for all possible 22 nt words would be 4^22^ = 1.76 × 10^13^ since there are four possible nucleotide letters at each of the 22 nt positions. The actual transcriptome contains much fewer than that number of possibilities (Table 1). Assume there are 5 × 10^4^ tRNA, rRNA, mRNA, and ncRNA different transcripts in a cell, with an average length of 2 × 10^3^ nt, then counting all overlapping words, there are 1 × 10^8^ possible words in a whole transcriptome matrix. This data set can fit on a big data scale computer system for analysis.

### 2.2. Simple Transcriptome Model

**Figure 1:**
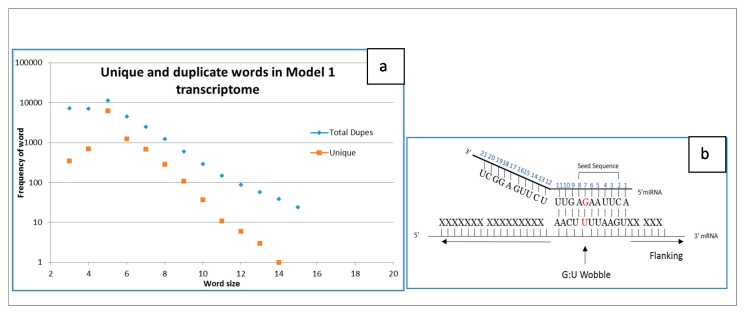
(**a**) Measures of number of unique and duplicate words in Simple Model transcriptome for various word size; (**b**) Correspondence to size of seed sequence for miRNAs. Red base pair is an allowed non-canonical matching.

As an approximation, we develop a transcriptome model version (simple model) using real highly expressed genes, and for comparison separately, randomized sequences of the transcriptome. The simplest realistic model is composed of 8 real human RNA transcripts as a simple representation of the transcriptome in a cell (Model 1). It is comprised of four of the most prevalent tRNAs with lengths of 71–73 nt (which happens to be slightly smaller than an average tRNA in Table 1), and four of the major subunits of the ribosome with sizes from 121 to 5034 nt. For this simple transcriptome, the total number of nucleotides is the sum of the nucleotides in each transcript, or 7470 nt. A program (TIC-generator) was written in C++ that calculates the frequency of words of length W that are contained in each transcript. For a RNA transcript of length L, the number of possible words would be L − W + 1. In the simple model, for each word length from W = 4 to 22, word count was calculated along with the sum of the frequencies of those words corresponding to the simple eight transcripts RNA = 1 to 8 labels (see Section 4). The output from TIC-generator is a listing of all words contained in each transcript, together with its frequency of occurrence. The lists of words from the eight transcripts were combined, and then duplicates removed. The number of duplicates and unique words resulting from duplicate removal is listed in Figure 1a. The total possible words of length W are 4^W^, shown as orange boxes in Figure 1a. The fraction of the possible words presents in the simple model transcriptome decreases for increasing word size. It is interesting that the peak in unique and total duplicate (blue diamonds in Figure 1b) words are maximal at the same size as the miRNA “seed” sequence. This peak seen in Figure 1a for duplicate words in a transcriptome construction would increase for increasing numbers of transcripts.

### 2.3. miRNA Datasets Examined with Simple Model Transcriptome

Experimental validation of the simple model transcriptome examined various functions of word similarity using published data sets. Functions tested include tWord for transcriptome words in common with target multiplied by word frequency in the transcriptome. Four studies below examined miRNA where data sources were grouped into high and low study parameter sets and mean values and *t*-test calculated with 2-tail *t*-test values under two-sample equal variance assumption models.

#### 2.3.1. Exosome Enriched miRNAs

**Figure 2:**
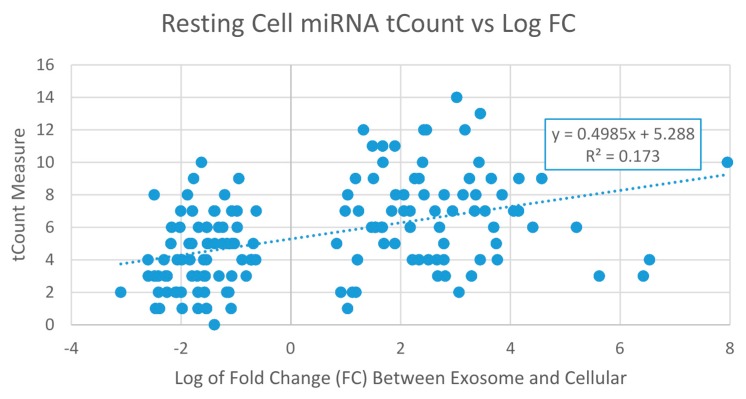
Resting miRNA cell tCount *vs.* Log fold change between exosome and cellular compartments. Word size was seven nt for calculation for tCount. Trendline added.

The Villarroya-Beltri [12] study performed microarrays on cellular and exosome fractions from resting and activated human T lymphocytes. They assessed whether certain RNAs are specifically classified into exosomes, and performed a microarray analysis of activation-induced variations in the miRNA and mRNA profiles of primary T lymphoblast and their exosomes. We used that data found in their Supplementary Data and data publicly available at Gene Expression Omnibus through GEO Series accession number GSE50972. Their microarray analysis showed that in most cases miRNAs modulated upon activation are different in cells and exosomes, either for upregulated or downregulated miRNAs. This shows that miRNA and mRNA loading into exosomes remains not a passive process. Certain miRNAs were more highly expressed in exosomes than in cells and in most circumstances this difference is preserved under resting and activated conditions. Similarly, most miRNAs that are more highly represented in cells than in exosomes keep this tendency free from the activation state of the cell. Then they classified some miRNAs as thus specifically sorted into exosomes (EXOmiRNAs), whereas others are specifically retained in cells (CLmiRNAs). We calculated the tCount of raw counts of words in common with the simple transcriptome and tWord, which factors the expression level of that word. Other measures compared tCount and tWord to a randomized transcriptome (RAN). We used a word size *w* = 7 roughly equal to the seed sequence length as shown in the peaks in Figure 1a,b. Figure 2 shows a clear tendency for the EXOmiRNA cluster on the right (average Log FC of 2.70) to be greater in value (average tCount of 6.80) than the CLmiRNAs (average Log FC of −1.62) in the left cluster of the data points with an average tCount of 4.32. A *t*-test between the two clusters gives a *p*-value of 3.2 × 10^−7^, indicating a significant difference between exosome and cytoplasmic miRNAs as measured with the tCount measure calculated from a simple transcriptome. To allow comparison with other classes of RNA, we can normalize the transcript size by dividing by sequence length. Transforming the tCount measure in Figure 2 increases the correlation coefficient to *R*^2^ = 0.185 with *y* = 0.0236*x* + 0.242, where *x* is log FC and *y* is tCount/Len for word size *w* = 7.

**Figure 3:**
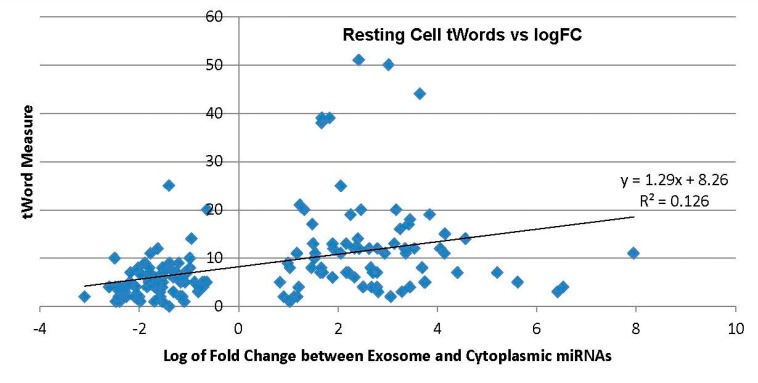
Resting cell miRNA tWords *vs.* Log fold change between exosome and cellular compartments. Trendline added.

For miRNAs with a resting LogFC, which was positive (average 2.70), values of tWord (mean 12.45) were higher than miRNAs with negative LogFC (average −1.62) for tWord (mean 5.47), and hence tWord was greater with miRNAs enriched in exosomes compared to cytoplasmic miRNAs in Figure 3.

A common pattern with tCount and tWord seen in Figure 2 and Figure 3 is the greater variance with exosomal miRNAs. Standard deviation of tCount is 32% greater in exosomal mrRNAs (S.D. = 2.9) compared to cytoplasmic (S.D. = 2.2). For the measure tWord, the difference is greater, with exosomal miRNAs having 168% greater standard deviation, 10.7 *vs.* 4.0. The greater variance with tWord compared to tCount is most likely due to the multiplier of expression level in the tWord calculation, as tCount is a simple count of occurrences of words in common between target miRNA and the transcriptome model.

**Figure 4:**
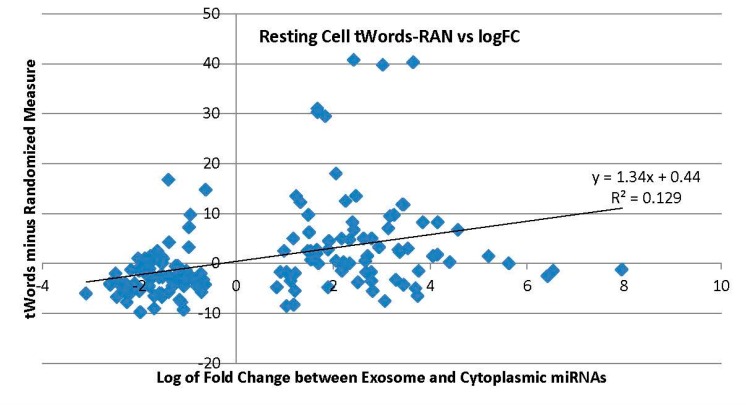
Resting cell miRNA tWords minus randomized sequence tWord score. Trendline added.

With eight outliers removed which had tWord scores above 25 in Figure 3, the new regression gives *y* = 0.85*x* + 6.88 and *R*^2^ = 0.182. This relationship is closely maintained even for activated cells, as with eight outliers removed gives *y* = 0.82*x* − 7.71 and *R*^2^ = 0.164. With six outliers removed for the function tWords-RAN, regression improves to *y* = 0.89*x* − 0.92 and *R*^2^ = 0.166 from the whole data set shown in Figure 4.

#### 2.3.2. Nuclear Enriched miRNAs

The Park (2010) study [13] compared nuclear and cytoplasmic fractions in hct116 colon cancer cells also by microarray. They recognized various miRNAs that existed in isolated nuclei from human colon cancer HC T116 cells. MicroRNA profiles were correlated between cytoplasmic and nuclear fractions of the HC T116 cell line by multiple microarray analyses. Nuclear confinement of the mature form of miRNAs was validated by controlling RT-PCR excluding the exposure of precipitate forms of miRNA, such as pri-miRNA or pre-miRNA. They established elevated levels of representative miRNAs in purified nuclei that support the notion that notable numbers of mature miRNAs survive not only in the cytoplasm but also in the nucleus.

Again we calculated tCount of raw counts of words in common with the simple transcriptome. The tWord factors the expression level of that word and other measures, comparing them to a randomized transcriptome (RAN). Their data was sorted by N/C ratio and partitioned into two groups: N/C > 0.47 which was nuclear enriched (*n* = 45), and N/C < 0.47 which are preferentially found in the cytoplasm (*n* = 33). tCount was 4.02 for nuclear enriched, and 5.00 for cytoplasmic, with a *t*-test *p*-value of 0.116 between the groups; while tWord was 4.73 for nuclear and 10.58 for cytoplasmic miRNAs, with a significant *t*-test *p*-value of 0.023 between nuclear and cytoplasmic groups. With this Park data set, dividing tCount and tWord by miRNA length yields improved *t*-test *p*-values of 0.094 and 0.022. Together this data suggests that nuclear-enriched miRNAs share fewer common words with the overall transcriptome than cytoplasmic miRNAs.

#### 2.3.3. Other miRNA Studies

The Huang (2013) study used RNA/seq on exosomes from human plasma [14]. The top 100 exosomes abundant miRNAs had tCount (mean 4.80) and tWord (mean 6.72) measures compared to those lower 100 with low “rcmm” reads (mean 4.64 and 7.41 respectively). Again, exosome transcripts have more similarity to the simple model transcriptome. Similar results are found with the Cheng (2014) study of exosomes in human blood [15]. There, the 50 most abundant miRNAs in exosome sampled labeled “Plasma UC Exo” had tCount and tWord values of 4.56 and 6.00 compared to 5.58 and 8.80 for low abundance transcripts. Related results also found with Guduric-Fuchs (2012) data on exosomes from HEK293 T cells showed that the ratio of EV to cell reads was significant whereas using read counts “rpmm” was not [16]. These data suggest that the relatedness of tCount and tWord measures to spatial partitioning are a function of enrichment factor and not abundance in the compartment.

As a step towards in-depth understanding the mechanism of selective exportation of miRNAs to EVs, Guduric-Fuchs (2012) employed deep sequencing to discriminate the global expression pattern of small RNAs in HEK293T cells and the EVs that they release [16]. Enrichment of overexpressed miRNA in EVs has been manifested by RT-qPCR in HEK293T cells, mesenchymal stem cells, macrophages and immune cells. Using data from Guduric-Fuchs that was sorted by EV/cell ratio, we compared the 10 top (exosome-enriched) and bottom (cytoplasmic enriched) miRNAs and listed tCount and tWord computed values in Table 2 as the mean and standard deviation in parenthesis. From Table 2 for the various measures examined across the studies, tCount, tWord and their difference (tW–tC), values progress from lower for nuclear, higher for cytoplasmic, and highest for exosomal miRNAs. Thus, under transitivity, EXO > CL > NUC for these transcriptome measures. This suggests that miRNAs with sequence similarity to the overall transcriptome transit furthest from their points of transcription. These conclusions are most significant with the tCount measure, with a *p*-value close to zero for the Villarroya-Beltri study, and 0.016 for the Guduric-Fuchs study, while the Park study showed little difference (*p*-value = 0.122) for tCount between nuclear and cytoplasmic enrichment.

## 3. Discussion

Much focus in RNA research is directed toward understanding the regulation of protein-coding genes [17]. However, ncRNAs also form well-orchestrated regulatory interaction networks [1]. For example, computational modeling of miRNA target sites suggests a broad network of miRNA-lncRNA interaction [18]. Recently, there have also been reports inferring the feasibility of a broad interaction network comprising competing endogenous RNAs (ceRNAs) where ncRNAs could change regulatory RNA by binding and titrating them off the corresponding binding sites on protein coding messengers [2].

We suggest that miRNA sequence delineates the molecular mechanisms underlying Brownian motion as a broad class of RNA with the transcriptome composed as an RNA language with interactions between transcripts and protein molecules at the same location. Recently, the attention of the relevant research community has been focused on non-coding RNAs and their physiological/pathological implications [19]. As the number of RNA experiments reported rapidly increases and transcriptional units are better annotated, databases indexing RNA properties and function from transcriptome measures become essential tools in this process. This early stage software development effort makes use of a sandbox-oriented software development environment [20] that enables development for miRNA physiology study. This work is generalizable to different sequence technologies, RNA/seq, microarray, *etc.*, and is scalable to different organisms [21], organs, or sub-cellular compartments depending on sample preparation for the libraries. Caution must be exercised with reported studies not adequately controlling for the source of extracellular particles, not differentiating between lipid coated RNAs, exosomes, microparticles, or apoptotic bodies.

## 4. Materials and Methods

### 4.1. Sliding Window Word Generator

**Figure 5:**
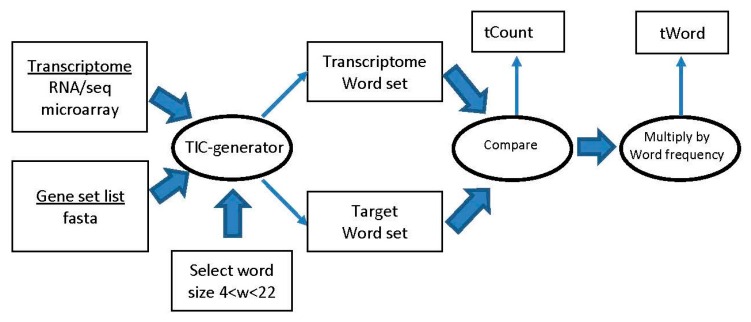
Logical workflow of transcriptome modeling.

A sliding window variable size word generator (TIC-generator) from input sequences was written in C++. A workflow functional diagram is shown in Figure 5. The output from TIC-generator is a listing of all words contained in each transcript, together with its frequency of occurrence. The lists of words from the eight transcripts were combined, and then duplicates removed. The number of duplicates and unique words resulting from duplicate removal is listed in Table 3. We provide program listing for the dissemination of software from the project in Supplementary Material, including software, tools and related resources, to the relevant research and user communities using open source resources.

### 4.2. Duplicate Words in Cloud

Eight transcripts were selected as representative of the most abundant RNAs in a cell. Four were tRNAs with lengths 71 and 73, and four were rRNAs with sizes 121, 156, 1871 and 5034 nucleotides. Individually within these eight transcripts, there is a total of 7470 nucleotides, which collectively have 7422 total words of length *w* = 7, with 1797 duplicate words leaving 5625 unique words describing the transcriptome as a simple model. If we combine the unique words of these eight transcripts, we find 691 duplicates, leaving a total of 4934 unique words in the simple model in Table 3. The combined total number of duplicates would be 1797 + 691 = 2488 while for the random transcriptome (average of four randomized transcriptomes) total duplicates are 840 + 659 = 1499. If instead we examine words of length *w* = 8, there are a total of 7400 words with 961 duplicates leaving 6439 unique words in the simple model. The combined total number of duplicates would be 961 + 288 = 1249 while for the random transcriptome (average of four randomized transcriptomes) total duplicates are 199 + 195 = 394.

## 5. Conclusions

miRNAs that are enriched in exosomes share greater similarity to the overall transcriptome than miRNAs found preferentially in the cytoplasm or nuclear compartments. Nuclear enriched miRNAs share less similarity to the transcriptome than cytoplasmic miRNAs. From the various measures examined in this study, tCount values progress from lower for nuclear, higher for cytoplasmic, and highest for exosomal miRNAs with the greatest significance.

## Figures and Tables

**Table 1:** Gross properties of typical Human Transcriptome.

Transcript Molecule	Size (nt)	Abundance (Copies)	Distinct Types	Notes
28S rRNA	5070	3.5 × 10^6^	1	Subunit in 80S ribosome
18S rRNA	1869	3.5 × 10^6^	1	Subunit in 80S ribosome
5.8S rRNA	156	3.5 × 10^6^	1	Subunit in 80S ribosome
5S rRNA	121	3.5 × 10^6^	1	Subunit in 80S ribosome
tRNA	~85	3 × 10^7^	~100	497 genes in 40 families, tissue specific
mRNA	2 kb	4 × 10^5^	4 × 10^5^	Tissue specific, many isoforms
ncRNA	>200	variable	>35,000	Complex isoforms [11]
miRNA	22	variable	1000	

**Table 2:** Various measures of word similarity to simple model transcriptome from 4 data sources.

Transcriptome Measures for Published Data Sets of miRNA										
Data Set	tCount	RANtCount	tWord	RANtWord	tW–tC	tC–RAN	tW–RAN	tC *Z*	tW *Z*	*N*
has-miR-	5.3 (2.9)	5.7	9 (10.9)	7.1 (1.9)	3.7 (9.0)	−0.4 (3.2)	1.9 (1.1)	−0.2 (2.4)	1.1 (7.3)	2588
V-B All	5.5 (2.9)	6.1 (1.3)	8.9 (8.8)	7.8 (1.9)	3.4 (6.8)	−0.6 (3.1)	1.1 (9.0)	−0.3 (2.0)	0.6 (4.6)	151
V-B EXO	6.8 (2.9)	6	12.5 (10.7)	7.7	5.7 (8.8)	0.8 (2.9)	4.8 (10.7)	0.4 (2.2)	2.2 (5.8)	75
V-B CL	4.3 (2.2)	6.3	5.5 (4.0)	8	1.2 (2.6)	−2.0 (2.7)	−2.4 (4.6)	−1.1 (1.5)	−1.0 (1.9)	76
Park All	4.4		7.2		2.8	−1.7	−0.4	−1.3	−0.3	78
Park NU	4.0 (2.0)		4.7 (2.6)		0.7 (1.1)	−2.3 (2.3)	−3.2 (2.9)	−1.6 (1.8)	−1.4 (1.4)	45
Park CL	5.0 (3.3)		10.6 (16.4)		5.6 (13.7)	−1.0 (3.7)	3.2 (16.8)	−0.8 (2.4)	1.1 (6.2)	33
G-F All	5.4 (3.5)		8.5 (8.6)		3.1 (6.0)					27
G-F EXO	7.9 (4.1)		13.5 (11.5)		5.6 (8.7)					10
G-F CL	4.0 (2.2)		6.1 (4.9)		2.1 (3.2)					10

Public database of miRNAs extracted 2588 human sequences. V-B from [12] with EXO exosome enriched or CL cytoplasmic miRNAs. Park from [13] with NUC nuclear enriched or CL cytoplasmic miRNAs. G-F from [16] with EXO exosome enriched or CL cytoplasmic miRNAs. Values in parenthesis are standard deviations. RANtCount and RANtWord are calculated from average of 4 randomized simple transcriptome words. tW-tC = tWord minus tCount and is a measure of the influence of frequent words in transcriptome. tC-RAN and tW-RAN are differences between tCount or tWord minus RANtCount or RANtWord, respectively. *Z*-scores from tCount and tWord calculated from RAN mean and SD of randomized simple transcriptome.

**Table 3:** Construction of transcriptome cloud for word size of 7 and 8.

*w* = 7	Transcript	1	2	3	4	5	6	7	8	Total	Unique	Duplicates
Transcript	nt length	73	73	71	71	156	121	5034	1871	7470		
	Word size											
Total words	*w* = 7	67	67	65	65	150	115	5028	1865	7422		
Unique words	*w* = 7	67	67	65	65	149	114	3355	1742	5625	4934	691
Duplicates	*w* = 7	0	0	0	0			1672	123	1797		
	Word size											
Total words	*w* = 8	66	66	64	64	149	114	5013	1864	7400		
Unique words	*w* = 8	66	66	64	64	149	114	4085	1831	6439	6439	288
Duplicates	*w* = 8	0	0	0	0	0	0	928	33	961		

Simple transcriptome model 1 based on 4 tRNAs (transcripts 1-4) and 4 subunits of the ribosome (transcripts 5–8).

## Supplementary Materials

Supplementary materials can be found at http://www.mdpi.com/1422-0067/17/6/830/s1.

## Abbreviations

miRNA	microRNA
nt	nucleotides
ceRNA	competitive endogenous RNA
ncRNA	non-coding RNA
